# Overexpression of lncRNA H19 enhances carcinogenesis and metastasis of gastric cancer

**DOI:** 10.18632/oncotarget.1913

**Published:** 2014-04-18

**Authors:** Hao Li, Beiqin Yu, Jianfang Li, Liping Su, Min Yan, Zhenggang Zhu, Bingya Liu

**Affiliations:** ^1^ Shanghai Key Laboratory of Gastric Neoplasms, Shanghai Institute of Digestive Surgery, Ruijin Hospital, Shanghai Jiao Tong University School of Medicine, Shanghai 200025, People's Republic of China.

**Keywords:** gastric cancer, long non-coding RNA, H19, microRNA, miR-675

## Abstract

Long non-coding RNAs (lncRNAs) play key roles in the progression and metastasis of some carcinomas. We previously showed that the expression of lncRNA H19 (H19) was higher in gastric cancer (GC) tissues than that in paired noncanerous tissues. However, the underlying mechanisms remain unclear.

In this study, H19/miR-675 knockdown models in the MKN45 cell line and ectopic expression models in the SGC7901 cell line were established, and a co-expression network of H19 was generated to identify target genes by RIP and DLR. The results showed that overexpression of H19 promoted the features of GC including proliferation, migration, invasion and metastasis. An H19 co-expression network identified ISM1 as a binding protein of H19, and its expression was positively correlated with that of H19. CALN1 was identified as a target gene of miR-675 and its expression was negatively correlated with that of miR-675. H19 and MiR-675 function in a similar manner. However, H19 RNA actively binds to ISM1 and miR-675 targets CALN1. These differences suggest that H19 plays other roles besides encoding miR-675 in GC. Our results suggest that the effect of H19 in GC is mediated by the direct upregulation of ISM1 and the indirect suppression of CALN1 expression via miR-675.

## INTRODUCTION

Gastric cancer (GC) is one of the most common cancers worldwide and its incidence is particularly high in East Asia and China. Approximately 988000 new cases of stomach cancer were reported in 2008 and the mortality rates in Eastern Asia were estimated at approximately 28.1 per 100,000 in men and 13.0 per 100,000 in women [1]. In China, the majority of GC patients are diagnosed at a late stage and their prognosis is poor. Therefore, elucidating the molecular mechanisms underlying GC progression is essential to identify key biomarkers and develop effective targeted therapies.

The human genome project suggested that only 1.2% of the mammalian genome encodes proteins [2-3], and most of the genome is transcribed to tens of thousands of long (>200 nt) non-coding RNAs (ncRNA) [4-5]. An increasing number of non-coding RNAs have been found to play critical roles in cancer development and metastasis [6-8]. For example, lncRNA HOTAIR in the HOX locus can increase the invasiveness and metastatic potential of human breast cancer by inducing genome-wide re-targeting of Polycomb Repressive Complex 2 (PRC2) to an occupancy pattern, which leads to histone H3 lysine 27 trimethylation and changes in gene expression [9]. MALAT-1, an abundant lncRNA in many human cell types, was suggested to regulate the alternative splicing of a subset of pre-mRNAs by modulating serine/arginine splicing factor activity, which regulates tissue or cell-type–specific alternative splicing in a phosphorylation dependent manner [10].

LncRNA H19 was discovered in 1991 by Bartolomei and shown to lack a common open reading frame in the RNA or an encoded protein. H19 is highly expressed in extraembryonic tissues, the embryo proper and most fetal tissues but its expression is dramatically reduced after birth [11-12]. Recent studies showed that H19 is overexpressed in several malignancies such as breast cancer [13-14], bladder cancer [15-16] and cervical carcinomas [17]. H19 expression is significantly correlated with tumor grade and is a marker of early recurrence in bladder cancer. Likewise, ectopic H19 expression enhances the tumorigenic potential of hepatocellular carcinoma cells in vivo [6], suggesting that H19 may have oncogenic properties in these types of cancers.

MiR-675 is a 23-nt RNA derived from nucleotides 1014−1036 of human H19 RNA [18]. Studies have shown that H19 can encode miR-675 and encoding miR-675 might be one of the important roles of H19 in human placental trophoblast cells and colorectal cancer cells [18-20]. However, whether H19 has any functions besides encoding miR-675 remains unknown. Whether the biological functions and co-expression network of H19 and miR-675 are the same is also unknown.

In the present study, we examined the expressions of H19 and miR-675 in GC and their relationship with clinicopathological factors. Furthermore, the roles of H19 and miR-675 in the proliferation, migration, invasion and metastasis were investigated in vitro and in vivo to gain a better understanding of their relationship and function in GC.

## RESULTS

### H19 is up-regulated in gastric cancer and correlated with poor prognosis

**Fig1:**
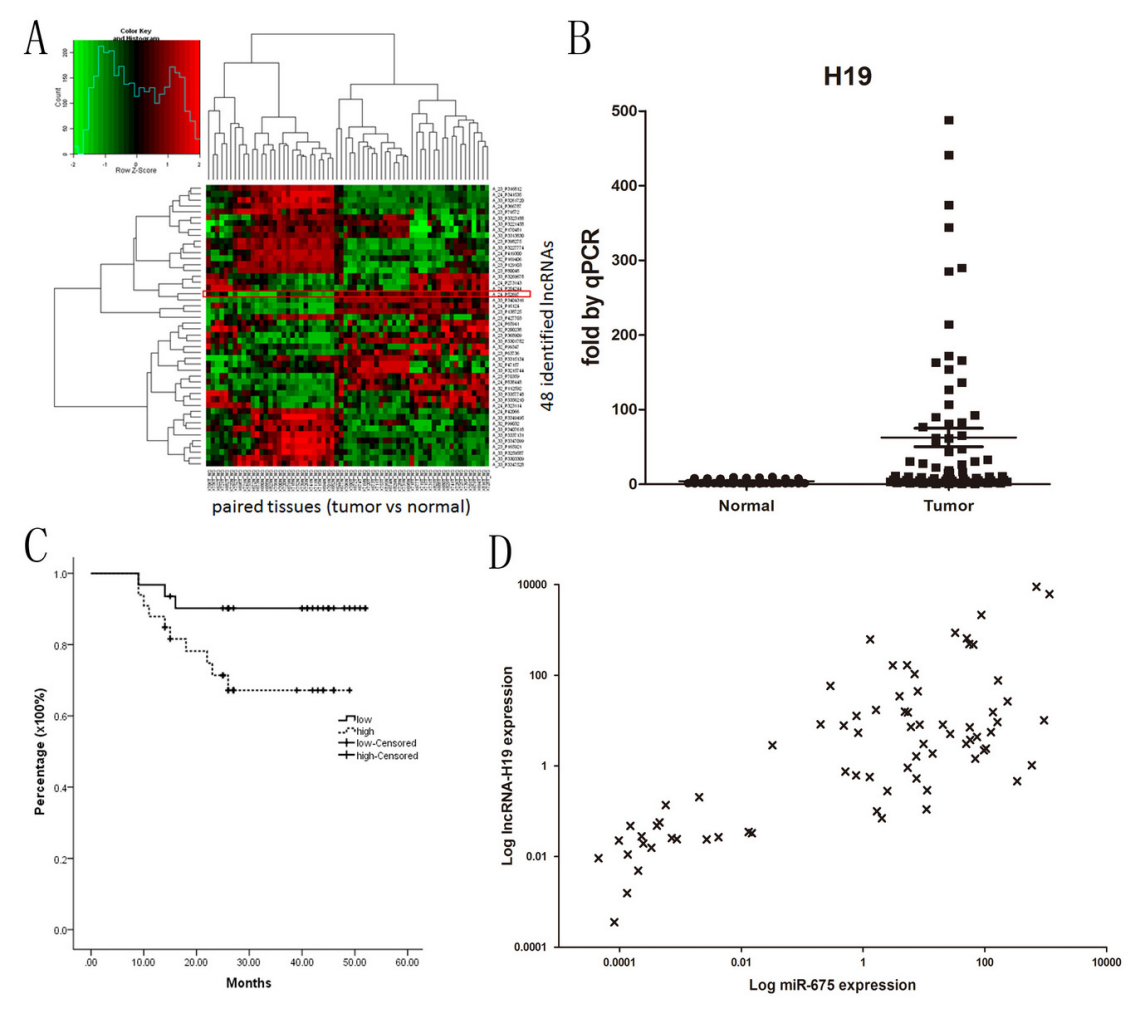
H19 and miR-675 are systematically up-regulated in gastric cancer and H19 has prognostic value for survival A) Heat map representing unsupervised hierarchical clustering of 48 lncRNAs expression values of a panel of 32 gastric cancers relative to paired noncancerous tissues. Each column represents the paired tissue samples. Each row indicates 48 candidate lncRNAs based on the 3 limitations at the 1^st^ screening. H19 is one such transcript (absolute fold change=6.6, *P*=0.0000361). B) Relative expression of H19 in 74 paired gastric cancer tumor tissues and noncancerous normal tissues. Statistical difference was analyzed by Wilcoxon signed–rank test (*P*=0.017). The relative expression of miR-675 showed the same pattern (data not shown). The H19 expression level was measured by quantitative reverse transcription-PCR. C) Kaplan-Meier curves for overall survival of the 74 patients with gastric cancer by H19 expression in tumor tissues (*P*=0.036). D) Positive correlation of H19 and miR-675 expression in 74 gastric cancer tissues.

The Agilent G3 Human GE Microarray (8×60K) was used to analyze lncRNA expression profiles in 32 GC tissues and paired noncancerous mucosae. The first screening identified 48 candidate lncRNAs that differed significantly between cancer and paired noncancerous tissues based on three limitations (Fig.1A) as follows: 1) Absolute fold change of lncRNA between cancer and paired noncancerous tissues higher than two; 2) Length of lncRNA shorter than 3 kb; 3) Inclusion of the lncRNA in the lncRNA database. In the second screening, we analyzed the expression of the 48 lncRNAs in GC cell lines and tissues by quantitative PCR and finally focused on H19 in our study.

Analysis of 74 paired clinical GC and noncancerous tissues showed a significant upregulation (mean: 6-fold) of the expression of H19 in cancer tissues compared with matched noncancerous tissues (Fig.1B). The 74 patients were then divided into two groups based on the fold-change of H19 expression as follows: H19-high (≥6-fold upregulation, n=36) and H19-low (<6-fold upregulation, n=38). In the univariate analysis, number of lymph nodes, metastasis, advanced TNM staging and H19 expression were significant prognostic factors (Table 1). However, no correlation between patient prognosis and age, gender, local invasion and Borrmann classification was observed. Pearson's correlation analysis showed that H19 expression was correlated with the number of lymph nodes and the clinical stage in our 74 patient samples (Tab.2). Kaplan-Meier survival analysis showed that patients with H19-high had a poorer prognosis than those with H19-low (*P*=0.036) (Fig.1C). These results suggested that a high H19 level is a significant predictor of death in GC.

**Tab 1:** Evaluate the prognostic factors by univariate analyses

Characteristic	Number	Percentage	Median SurvivalTime (Months)	P value
Gender				
Male	54	73.0%	26 (1-51)	0.187
Female	20	37.0%	41 (2-53)	
Age				
<60	33	44.6%	26 (8-53)	0.837
≥60	41	55.4%	27 (1-53)	
Primary tumor				
T1-2	19	25.7%	40.5 (8-53)	0.194
T3-4	55	74.3%	26 (1-52)	
Lymph nodes				
N0-1	30	40.5%	40 (8-53)	0.027
N2-3	44	59.5%	26 (1-53)	
Metastasis				
M0	70	94.6%	27 (1-53)	0.001
M1	4	5.4%	10 (2-14)	
Clinical stage				
I-II	33	44.6%	40.5 (8-53)	0.001
III-IV	41	55.4%	26 (1-53)	
Borrmann classification				
I-II	57	77.0%	26.5 (1-53)	0.754
III-IV	17	23.0%	25.5 (8-48)	
Fold of H19 Expression				
Low (<6)	38	51.4%	40 (1-53)	0.003
High (≥6)	36	48.6%	25 (2-49)	

**Tab 2:** Pearson Correlation analysis

Characteristic	Number	Percentage	Fold of H19 (P value)	Fold of miR-675
Male	54	73.0%	0.360	0.429
Female	20	27.0%		
Age				
<60	33	44.6%	0.672	0.799
≥60	41	55.4%		
Primary tumor				
T1-2	19	25.7%	0.531	0.365
T3-4	55	74.3%		
Lymph nodes				
N0-1	19	25.7%	0.029(0.254)	0.139
N2-3	55	74.3%		
Metastasis				
M0	70	94.6%	0.388	0.901
M1	4	5.4%		
Clinical stage				
I-II	33	44.6%	0.019(0.272)	0.075
III-IV	41	55.4%		
Borrmann classification				
I-II	57	77.0%	0.789	0.211
III-IV	17	23.0%		

We also found that miR-675 expression was upregulated in tumor tissues compared to the paired noncancerous tissues. Furthermore, a positive correlation between H19 and miR-675 expression was observed in GC tissues (r=0.558, *P*=0.002) (Fig.1D) and cell lines, confirming that miR-675 is derived from H19.

### H19/miR-675 upregulation promotes cell proliferation in vitro

**Fig2:**
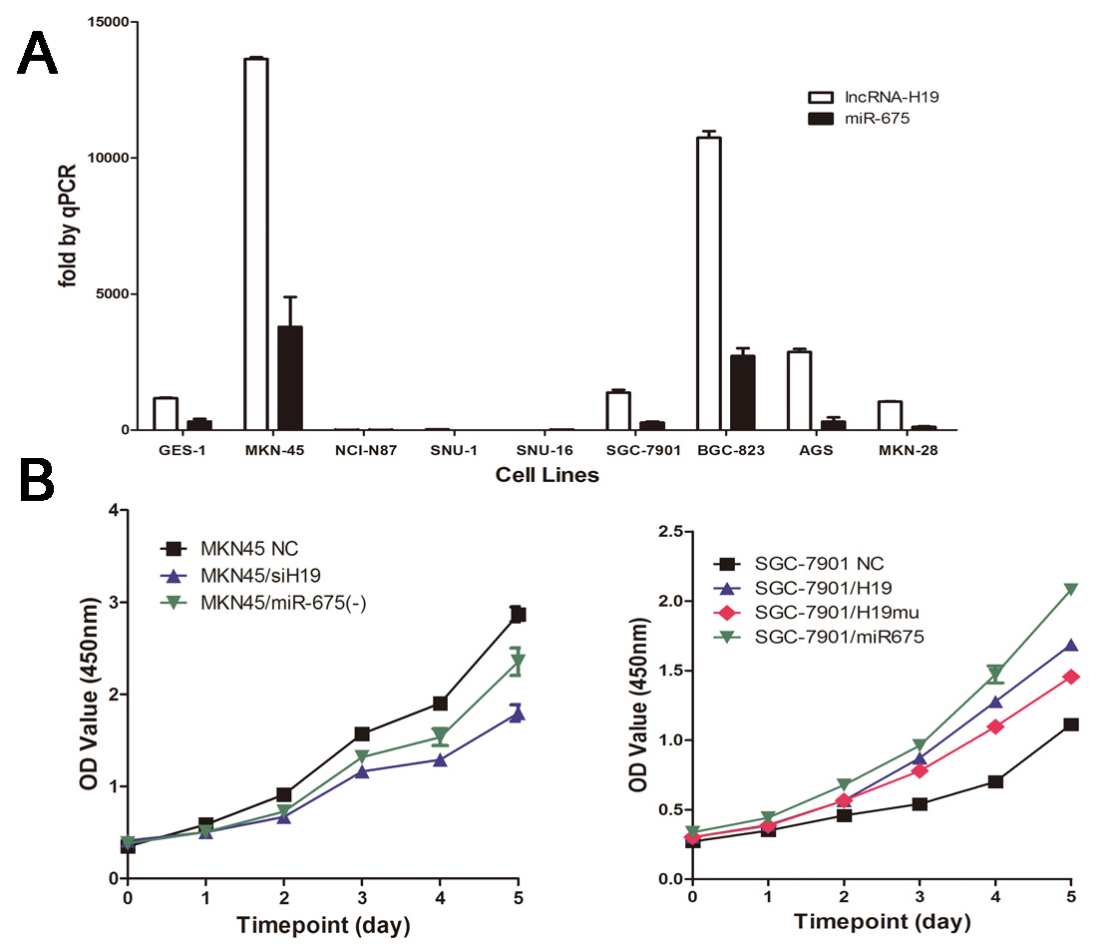
H19 and miR-675 promote cell proliferation of gastric cancer cells A) Relative expression of H19 and miR-675 in human gastric cancer cell lines and human immortalized gastric epithelial cell line (GES-1) measured by quantitative reverse transcription-PCR. B) Cell proliferation was measured by CCK-8 assay. MKN45 cells were knock-down H19 or miR-675 expression by siRNA respectively (left panel). SGC7901 cells were transfected with H19 or miR-675 as well as H19 with 1^st^ exon depletion (H19mu) (right panel). The CCK-8 assay was performed every 24h for 5 days and the results were means of sextuplicate.

Analysis of the expression of H19 and miR-675 in different GC cell lines showed that H19 was expressed at high levels in MKN45 and BGC-823 cells and at low levels in the NCI-N87, SNU-1, SNU-16, SGC7901, and MKN28 lines. We then selected MKN45 cells for knock-down experiments and SGC7901 cells for overexpression experiments (Fig. 2A). H19 expression was silenced in MKN45 cells by transfection with a specific siRNA and overexpressed in SGC7901 cells by transfection with the full length H19. H19 knock-down inhibited cell proliferation compared to the non-transfected controls (NC), whereas H19 overexpression had the opposite effect (Fig. 2B). Similar results were obtained in response to miR-675 knock-down or overexpression in GC cell lines. However, the cell proliferation curves for H19 and miR-675 did not overlap. Significant differences in the cell numbers from day 3 to day 5 were observed between *SGC7901/H19* and *SGC7901/miR-675* (*P*<0.05). These results indicated that the effect of miR-675 in GC cannot fully compensate for the effect of H19.

To determine whether H19 has other functions in addition to encoding miR-675, a mutant H19 transcript (H19mu) was developed by deletion of the first exon and transfected into SGC7901 cells to generate *SGC7901/H19mu*. Overexpression of H19mu promoted the growth of *SGC7901/H19mu* cells compared with the NC group (Fig.2B), indicating that the effect of H19 on increasing GC cell proliferation is not only mediated by miR-675.

### H19/miR-675 upregulation promotes cell migration and invasion in vitro

**Fig3:**
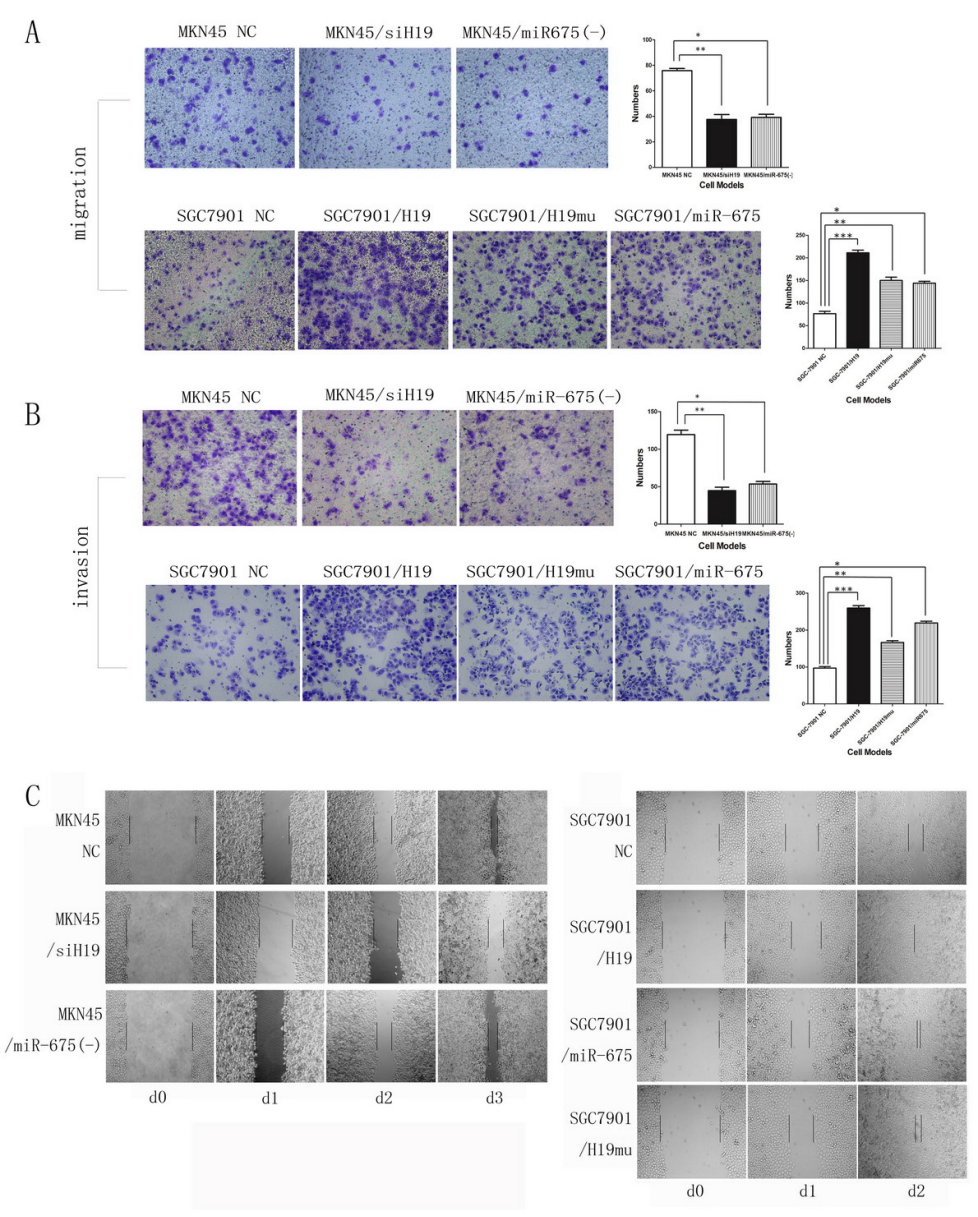
H19/miR-675 promotes migration and invasion of MKN45 or SGC7901 cells based on transwell and wound-healing assay A) Representative photographs of migratory cells on the membrane (magnification, 100x). B) Representative photographs of invasion cells on the membrane (magnification, 100x). The right panel of each row in A and B is the average cell number of triplicate (*, **, *** p<0.05). MKN45 cells were knock-down H19 or miR-675 expression (upper panel). SGC7901 cells were transfected with H19 or miR-675 as well as H19 with 1^st^ exon depletion (H19mu) (lower panel). C)The rate of migration was measured by quantifying the total distance from the edge of the wound toward the other side (distance between two lines) for 2 or 3 days.MKN45 cells were knock-down H19 or miR-675 expression (left panel). SGC7901 cells were transfected with H19 or miR-675 as well as H19 with 1^st^ exon depletion (H19mu) (right panel).

We further assessed the effects of H19 and miR-675 on cell migration and invasion, which are key determinants of malignant progression and metastasis. As shown in Fig. 3, overexpression of H19 (*SGC7901/H19*) or miR-675 (*SGC7901/miR-675*) significantly increased migration and invasion of SGC7901 cells in the Transwell assay. By contrast, migration and invasion were significantly decreased in the MKN45 knock-down group (*MKN45/siH19* and *MKN45/miR-675(-)*). These results suggest a functional role for H19 and miR-675 in mediating cell migration and invasion in GC and reveal a mechanism by which the up-regulation of H19 and miR-675 may contribute to tumor metastasis in GC. In addition, significant differences between H19 and miR-675 were detected in the migration and invasion assay (Fig. 3 A and B), suggesting that H19 and miR-675 have different target proteins and act via different signaling pathways.

In the wound-healing assay, the scratch healed faster in SGC7901 cells overexpressing H19 (*SGC7901/H19*) or miR-675 (*SGC7901/miR-675*) than in the NC group. Conversely, healing was slower in MKN45 cells with knocked down H19 (*MKN45/siH19*) or miR-675 (*MKN45/miR-675(-)*) than that in NC (Fig. 3C).

Compared with *SGC7901/NC* in the overexpression group, upregulation of H19mu (*SGC7901/H19mu*) also increased the migration and invasion of SGC7901 cells in the Transwell and wound-healing assays (Fig. 3), indicating that H19 promotes GC cell migration and invasion via other mechanisms besides encoding miR-675.

### H19/miR-675 upregulation promotes tumor igenesis and metastasis in vivo

**Fig4:**
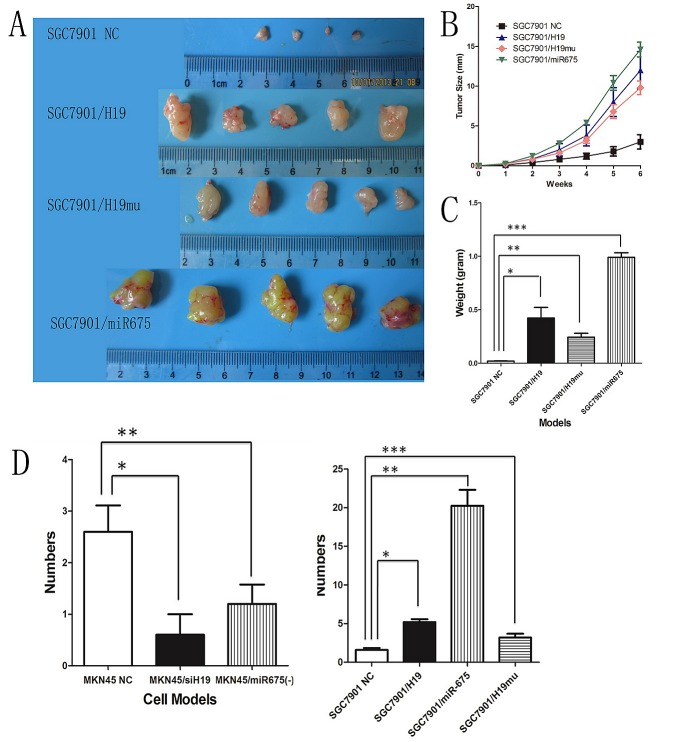
H19 or miR-675 enhances tumor growth and metastasis in vivo A) Photographs of tumors derived from SGC7901 cells six weeks after transfected with H19, miR-675 and H19mu, respectively. B) The tumor growth curves of SGC7901 ectopic expression subgroups showed the same pattern as cell proliferation assay. C) Average weight of tumors derived from SGC7901 cells six weeks after transfected with H19, miR-675 and H19mu, respectively. D) Average number of peritoneal metastatic nodules in H19/mioR-675 expression knock-down of MKN45 cells (left panel) and H19/miR-675 and H19mu ectopic expression of SGC7901 cells (right panel).

Ectopic expression of H19 increased growth, migration and invasion of GC cells in vitro. Therefore, we examined whether overexpression of H19 could enhance tumor growth and metastasis in vivo by subcutaneously injecting *SGC7901/H19* cells into nude mice and monitoring tumor growth weekly. After 6 weeks, the mice were euthanized and tumors were weighed. The results showed that tumors grew faster in *SGC7901/H19* transfected mice than that in the NC group (Fig.4B). Furthermore, tumor growth curves showed exactly the same pattern as that observed in the overexpression cell models in vitro. The tumor diameters of *SGC7901/H19* transfected mice were significantly larger than those of the NC group on week 6 (Fig. 4A). The average tumor weight of mice inoculated with H19-transfected SGC7901 cells was 180.6 ± 62.9 mg, which was significantly higher than that of the NC group (476.1 ± 55.6 mg; P < 0.05, Fig. 4C). In addition, we tested whether overexpression of miR-675 (*SGC7901/miR-675*) or H19mu (*SGC7901/H19mu*) could enhance tumor growth in vivo and obtained similar results, showing that both *SGC7901/miR-675* and *SGC7901/H19mu* cells grew faster than *SGC7901/NC*.

Injection of *SGC7901/H19mu* cells into nude mice showed that the subcutaneous tumors grew faster and tumor weights were significantly higher on week 6 than those of *SGC7901/NC* mice.

*SGC7901/H19* and *SGC7901/miR-675* cells were injected into the peritoneal cavity of 4-week-old female nude mice. Eight weeks after injection, the mice were euthanized, and the peritoneal metastatic nodules were counted. The average numbers of peritoneal metastatic nodules in *SGC7901/H19* or *SGC7901/miR-675* mice were significantly greater than those of *SGC7901/NC* mice (1 ± 0.7 vs. 3.8 ± 0.8, *P*< 0.05) (Fig. 4D). Intraperitoneal injection of 4-week-old female nude mice with *MKN45/siH19* and *MKN45/miR-675(-)* cells showed that compared with *MKN45/NC*, knock-down of H19 or miR-675 expression decreased the number of peritoneal metastatic nodules (Fig.4D). Knock-down of H19 was more efficient than that of miR-675 in inhibiting peritoneal metastasis in vivo. Taken together, these results suggested that H19 promotes tumorigenicity and metastasis not only via encoding miR-675 but potentially via other mechanisms, which is consistent with the data obtained in the proliferation, migration and invasion assays in vitro.

### H19 regulates the expression of its binding protein ISM1

**Fig5:**
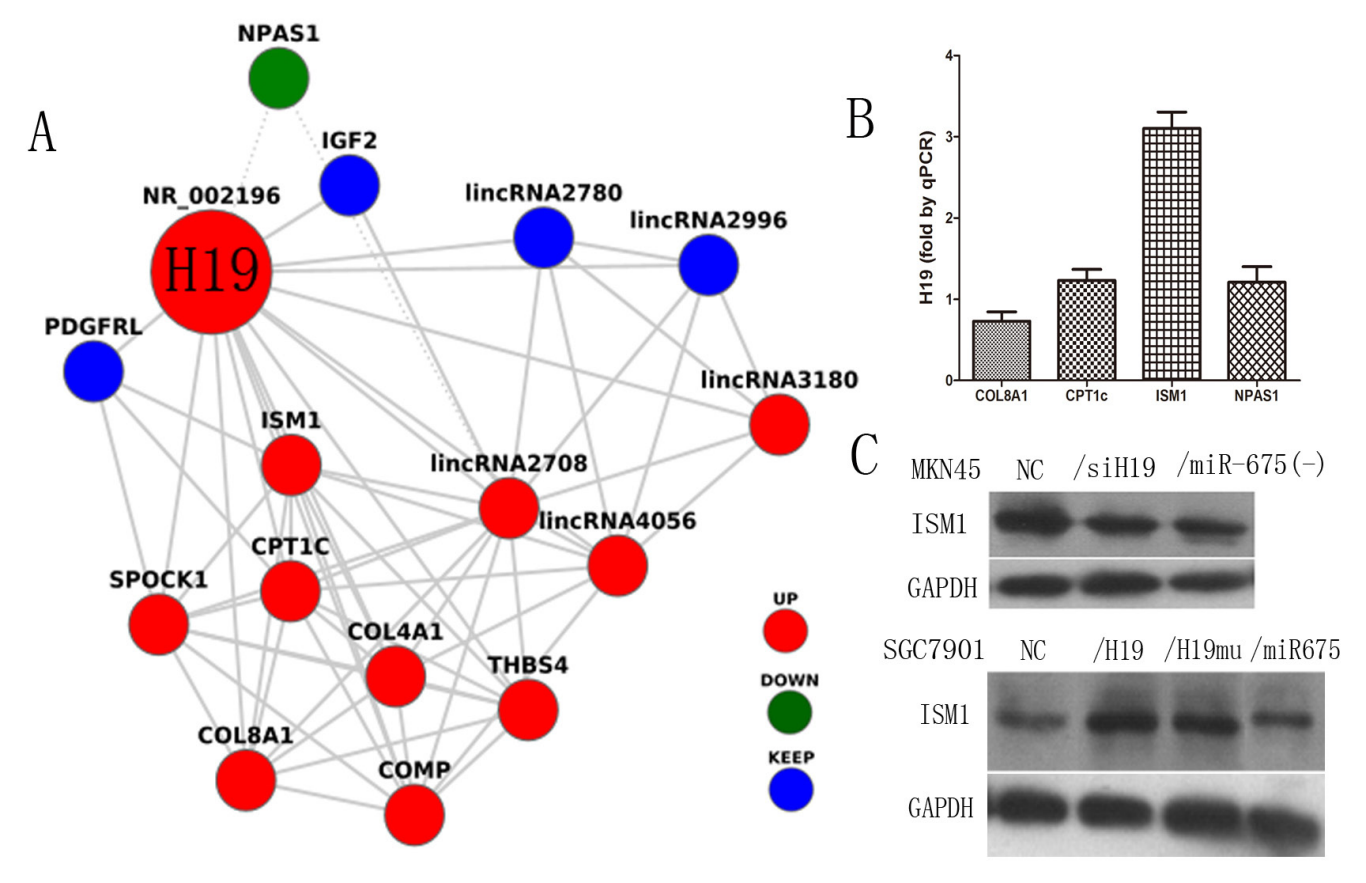
Co-expression network of H19 and the identification of the H19 binding protein A)Photograph of the H19 co-expression network was built on the data from microarray and identified by the Gene Ontology (GO) analysis and Kyoto Encyclopedia of Genes and Genomes (KEGG) database (up-regulated: full lines, down-regulated: dotted lines). B) The expression of H19 in the RBP Immunoprecipitation by RIP assay measured by quantitative reverse transcription-PCR. C) The ISM1 protein level in H19/miR-675 knock-down of MKN45 cells and ectopic expression of SGC7901 cells.

Bonferroni multiple testing correction was applied to the enrichment *P*-value (*P*≤0.01) in the functional annotation categories based on genes identified in the microarray [21-22]. We used Gene Ontology (GO) analysis and the Kyoto Encyclopedia of Genes and Genomes (KEGG) database to identify the biological functions and pathways mediated by differentially expressed genes associated with H19 and built a co-expression network for H19 (Fig. 5A). Of three candidate proteins selected in the co-expression network, ISM1 was identified as the binding protein of H19 by RNA-binding protein immunoprecipitation (RIP) followed by qPCR in MKN45 cells (Fig.5B). Furthermore, ISM1 protein expression was examined in all knock-down and overexpression subgroups by western blotting, which showed that ISM1 was expressed at higher levels in MKN45 cells than in *MKN45/siH19* and *MKN45/miR-675(-)* cells (Fig. 5C). Ectopic expression of H19 in SGC7901 cells upregulated ISM1 expression compared to that in *SGC7901/NC* cells (Fig. 5C). These results indicated that ISM1 is a binding protein of H19 and its expression is regulated by H19.

### CALN1 is a target of miR-675

**Fig6:**
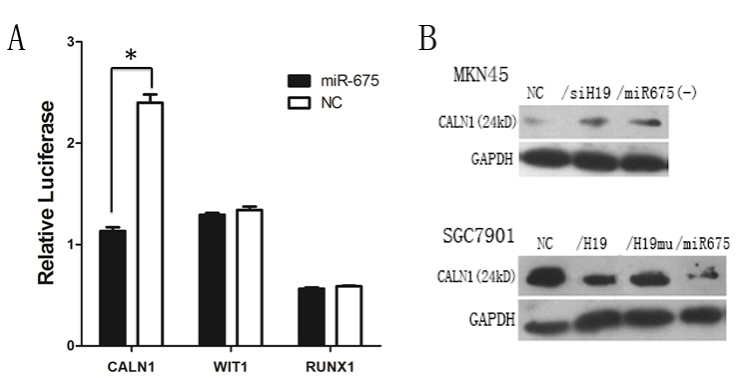
miR-675 targets CALN1 expression A) Relative luciferase activity of CALN1construct with miR-NC was significantly increased, however, the changes in the luciferase activity of WIT1 and RUX1 were not observed. B) The CALN1 protein level in H19/miR-675 knock-down of MKN45 cells and ectopic expression of SGC7901 cells.

The targets of miR-675 were predicted by searching and combining the results from two microRNA databases, TargetScan 6.2 and mirDB. Three candidate targets were identified, CALN1, WIT1 and RUNX1, and the Dual-Luciferase Reporter Assay was used to confirm the target for miR-675. The 3'-UTR of the three candidates' mRNAs flanking the entire putative target sequence or a 3'-UTR with a mutated target sequence as miR-NC were subcloned into the firefly luciferase reporter vector (psiCHECK-2 vector). The construct was then co-transfected with miR-NC into T293 cells. Our results showed that the relative luciferase activity of the psiCHECK-CALN1 construct with miR-NC was significantly increased in T293 cells, and overexpression of miR-675 significantly decreased the relative luciferase activity in T293 cells (*P*=0.0002). However, no changes in the luciferase activities of psiCHECK-WIT1 and psiCHECK-RUX1 were observed upon transfection with miR-675 or miR-NC (Fig. 6A). Assessment of CALN1 protein levels in response to the overexpression or silencing of miR-675 showed that downregulation of miR-675 increased CALN1 protein levels in GC (Fig.6B), whereas ectopic miR-675 expression consistently decreased CALN1 protein levels in our SGC7901 overexpression models (Fig.6B). Taken together, the data from the luciferase activity assay and western blot analysis strongly support that CALN1 is a direct target of miR-675.

## DISCUSSION

The mechanisms underlying the role of lncRNAs in transcriptional regulation are complex [23-24]. Studies have suggested that the oncogenic role of H19 is associated with its function as the precursor of miR-675 [17,19]. However, the underlying mechanism remains unclear. In the present study, we investigated the involvement of H19 and miR-675 in GC and showed that H19 has other functions besides encoding miR-675. The roles of H19 independent of its association with miR-675 do not overlap.

Our data showed that both H19 and miR-675 were expressed at higher levels in GC tissues than in noncancerous tissues, and H19 expression was positively correlated with lymph node metastasis and clinical stage. High expression of H19 in GC was correlated with poor prognosis based on our survival analysis. However, no correlation was observed between miR-675 expression in GC and pathological parameters.

As miR-675 is derived from the first exon of H19, a first exon deletion mutant of H19 was used to examine whether H19 has other functions besides encoding miR-675. Our data showed that H19mu promoted cell proliferation, migration and invasion in vitro and increased tumorigenesis and metastasis in vivo However, our data also showed that the functions of H19 and miR-675 do not entirely overlap. These results suggested that both H19 and miR-675 have oncogenic roles in GC and H19 has other functions in addition to its role as the precursor of miR-675.

H19 RNA may interact directly with its target genes or indirectly through specific H19 interaction proteins [6,25]. In our study, ISM1 was identified as the target gene of H19, whereas CALN1 was identified as the target of miR-675. Our results showing that H19 and miR-675 have different targets suggest that they play different roles during gastric cancer development and that they act through different pathways. Further exploration of the roles and regulation of the H19/ISM1 and miR-675/CALN1 pathways would be of interest in future studies.

In summary, we showed that H19 and its associated miR-675 act as oncogenes by promoting cell growth and malignant transformation in human gastric cancer. The upregulation of H19 and miR-675 in GC suggests that both H19 and miR-675 are important factors in GC tumorigenesis and metastasis. Furthermore, we showed that H19 plays additional roles mediated by separate pathways and interaction with its target gene ISM1 that are independent from its association with miR-675. Our results indicate that H19 may serve as a potential prognostic marker and therapeutic target for the treatment of GC.

## MATERIALS AND METHODS

### Patient samples

A total of 106 gastric cancer patients who underwent D2 radical gastrectomy between 2009 and 2011 in our hospital (Shanghai RuiJin Hospital, Shanghai Jiao Tong University, School of Medicine) were enrolled in the study. All the specimens including cancer and paired noncancerous tissues were placed in liquid nitrogen with RNALater (Qiagen) immediately after resection and stored until RNA extraction. A total of 32 paired specimens were used for microarray analysis (Agilent G3 Human GE 8×60K Microarray) and the other 74 paired specimens were used for identification and clinical follow-up. The median follow-up was 27 months (range, 9−53 months). The clinical staging criteria were obtained from the AJCC TNM Classification edition 7 in 2010 and the clinicopathological data of the cohort are shown in Table 1.

### Cell lines

The human gastric epithelial cell line GES-1 and the gastric cancer cell lines SGC7901, MKN45, MKN-28, NCI-N87, BGC-823, SUN-1, SUN-16, and AGS were purchased from American Type Culture Collection (Manassas, VA) and Shanghai Institute of Cell Biology (Shanghai, China). All the cell lines were maintained routinely in RPMI Media 1640 (Gibco, Cat#:11875-093) supplemented with 10% fetal bovine serum and grown at 37°C in a 5% CO2 atmosphere.

To build the H19 and miR-675 overexpression SGC7901 cell models, the full-length H19 cDNA and miR-675 precursor were transfected into SGC7901 cells to generate *SGC7901/H19* and *SGC7901/miR-675*, respectively. H19 cDNA with a deletion mutation in the first exon was transfected into SGC7901 cells to generate *SGC7901/H19mu*.

Knock-down H19 and miR-675 models in MKN45 cells were developed by transfecting siRNAs against H19 and miR-675 into MKN45 cells to generate *MKN45/siH19* and *MKN45/miR-675(-)*, respectively. Empty lentiviral vectors were transfected as negative controls and termed *SGC7901/NC* and *MKN45/NC*, respectively.

### RNA Extraction and qRT-PCR

Total RNA was extracted from tissues or cells using the Trizol reagent (Invitrogen) according to the manufacturer's instructions. Total RNA was eluted with RNase-free water and stored at -80°C. RNA concentrations were determined by Epoch spectrophotometry.

The quantitative real-time polymerase chain reaction (PCR) was performed by using SYBR-green PCR Master Mix in a Fast Real-time PCR 7500 System (Applied Biosystems). The gene-specific primers were as follows: H19 (forward: 5'-ATCGGTGCCTCAGCGTTCGG-3'; reverse: 5'-CTGTCCTCGCCGTCACACCG-3'); miR-675 (forward: 5'-CCCAGGGTCTGGTGCGGAGA-3'; reverse: 5'-CAGGGGCTGAGCGGTGAGGG-3'). β-Actin was amplified in parallel as the internal control. PCR reactions were performed at 50°C for 2 min, followed by 40 cycles of 95°C for 15 s and 60°C for 1 min. ΔCt was calculated by subtracting the Ct of U6 or β-actin RNA from the Ct of miR-675 or the mRNA of interest, respectively. ΔΔCt was then calculated by subtracting the ΔCt of the control from the ΔCt of the treatment group. Fold change of H19 or miR675 was calculated by the equation 2-ΔΔCt.

### Ectopic expression and knockdown of H19, miR-675 and H19mu

Based on the expression of H19 and miR-675 in GC cell lines, we selected SGC7901 cells for the enhanced expression study and MKN45 cells for the knock-down study. For ectopic expression, the full-length H19 cDNA and miR-675 precursor were subcloned into the GV299 lentiviral vector (Genechem, Shanghai) and transfected into SGC7901 cells to generate *SGC7901/H19* and *SGC7901/miR-675*. The H19 cDNA with a deletion mutation of its first exon was subcloned into the GV299 lentiviral vector and transfected into SGC7901 cells to generate *SGC7901/H19mu*.

For knock-down of H19 expression, two complementary oligonucleotides of small hairpin RNAs targeting the 5′-CGTGACAAGCAGGACATGA-3′ sequence were chemically synthesized, subcloned into the GV115 lentiviral vector (Genechem, Shanghai) and transfected into MKN45 cells to generate MKN45/siH19. Knock-down of miR-675 was performed by transfection with anti-miR-675 (Genechem, Shanghai) and then transfected into MKN45 cells to generate MKN45/miR-675(-).

Briefly, A total of 1.2x10^7^ cells were plated in 15 cm culture dishes for 24 h and then transfected with the vectors described above using Lipofectamine 2000 (Invitrogen) for 24 h. The negative control (Genechem, Shanghai) was transfected in parallel. The cells were then subjected to RNA/protein extraction and further functional assays

### Cell proliferation assay

Cell proliferation was monitored by a colorimetric assay using water-soluble tetrazolium salt (WST-8) and the Cell Counting Kit-8 (Dojindo) according to the manufacturer's instructions. Briefly, 100 μl of cell suspension from each subgroup (2,000 cells/well) was placed in a 96-well plate and pre-incubated for 12 h. Then, 10 μl of CCK-8 solution was added to each well and the number of cells was counted every 24 h for 5 days by measuring the absorbance at 450 nm using Epoch Microplate Spectrophotometer (Bio Tek).

### Cell migration and invasion assay

Cell migration was assessed using Control Cell Culture Inserts in two 24-well plates of 8 μm (BD Biosciences) according to the manufacturer's instructions. Briefly, 200 μl of serum-free medium containing 2×10^5^ cells from each subgroup were add to the upper chamber. A volume of 0.6 ml of 20% FBS-containing medium was then added to the lower chamber as a chemoattractant. Cells were incubated for another 16 h at 37°C in 5% CO_2_.

For the invasion assay, Matrigel Invasion Chambers in two 24-well plates of 8 μm (BD Bioscience) were used according to the manufacturer's instructions. Briefly, 200 μl of serum-free medium containing 1×10^5^ cells from each subgroup were add to the upper chamber. A volume of 0.6 ml of 20% FBS-containing medium was then added to the lower chamber as a chemoattractant. Cells were incubated for another 40 h at 37°C in 5% CO_2_.

After the incubation, cells on the upper surface of the membrane were scraped off with cotton swabs. Cells migrated to the bottom of the membrane were fixed and stained with 0.1% Crystal Violet Staining Solution. The cells on the bottom of the membrane were counted from five different microscopic fields and the average number was calculated.

### Wound-healing assay

The wound healing assay allows the researcher to study cell migration and cell interactions. A total of 1.5x10^6^ cells were seeded in 6-well plates and cultured overnight until confluent. A (yellow) pipette tip was used to make a straight scratch. The suspension cells were washed off twice gently and images of the scratch were acquired as baseline. The medium was then replaced and images of the same location were obtained every 24 h for the next 5 days.

### RNA Binding Protein Immunoprecipitation

The RIP assay was performed using the Magna RIP™ RNA-Binding Protein Immunoprecipitation Kit (Millipore) according to the manufacturer's instructions. Briefly, cells were harvested and lysed in RIP Lysis Buffer. RNAs were immunoprecipitated with an antibody against RBP and protein A/G magnetic beads. The magnetic bead bound complexes were immobilized with a magnet and unbound materials were washed off. Then, RNAs were extracted and analyzed by qRT-PCR.

### Luciferase activity assay

T293 cells were cultured in 6-well plates and transfected with 100 ng of psiCHECK-CALN1 or psiCHECK-WIT1 or psiCHECK-RUNX1 vectors containing firefly luciferase together with 50 ng of miR-675 or control. Transfection was performed using Lipofectamine 2000 (Invitrogen). At 24 h post-transfection, relative luciferase activity was calculated by normalizing the Firefly luminescence to the Renilla luminescence using the Dual- Luciferase Reporter Assay (Promega) according to the manufacturer's instructions.

### Western blot analysis

Cells in culture were lysed using the RIPA buffer (Pierce) in the presence of a Protease Inhibitor Cocktail (Pierce). The protein concentration of the lysates was measured using the BCA Protein Assay Kit (Pierce). Equivalent amounts of protein were resolved and mixed with 5× Lane Marker Reducing Sample Buffer (Pierce), electrophoresed in 12.5% SDS polyacrylamide gels and transferred onto Immobilon-P Transfer Membranes (Millipore). The membranes were blocked with 5% non-fat milk in Tris-buffered saline and then incubated with antibodies followed by horseradish peroxidase-conjugated secondary antibody (Abcam). Signals were detected with Immobilon Western chemiluminescent HRP Substrate (Millipore). GAPDH (Abcam) was used as a loading control.

### Tumor xenograft model and tumorigenicity assay

Stably transfected cells (1×10^6^ cells/mouse, 0.2 ml) were subcutaneously injected into 5-week-old male nude mice. The mice were euthanized 6 weeks after injection, and the tumors were removed and weighed.

### Metastasis assay in vivo

Stably transfected cells were injected into the abdominal cavity of 5-week-old male nude mice (2×10^6^ cells/mouse, 0.2 ml). Eight weeks after injection, the mice were euthanized and the number of metastatic nodules was documented.

### Statistical analysis

The relationships between H19 and miR-675 expression levels and clinicopathologic parameters and overall survival were analyzed by univariate analyses, Pearson's correlation analysis and Kaplan-Meier survival analysis. When comparisons were made between two different groups, statistical significance was determined using the Student's t test. All statistical analyses were performed using IBM SPSS Statistics v19 software package. A two-tailed value of *P*< 0.05 was considered statistically significant.

## References

[1] International Agency for Research on Cancer (IARC)

[2] ENCODE Project Consortium (2007). Identification and analysis of functional elements in 1% of the human genome by the ENCODE pilot project. Nature.

[3] FANTOM Consortium; RIKEN Genome Exploration Research Group and Genome Science Group (Genome Network Project Core Group) (2005). The transcriptional landscape of the mammalian genome. Science.

[4] Johnson JM, Edwards S, Shoemaker D, Schadt EE (2005). Dark matter in the genome: evidence of widespread transcription detected by microarray tiling experiments. Trends Genet.

[5] Furuno M, Pang KC, Ninomiya N, Fukuda S, Frith MC, Bult C, Kai C, Kawai J, Carninci P, Hayashizaki Y, Mattick JS, Suzuki H (2006). Clusters of internally primed transcripts reveal novel long noncoding RNAs. PLoS Genet.

[6] Matouk IJ, DeGroot N, Mezan S, Ayesh S, Abu-lail R, Hochberg A, Galun E (2007). The H19 non-coding RNA is essential for human tumor growth. PLoS One.

[7] Whitehead J, Pandey GK, Kanduri C (2009). Regulation of the mammalian epigenome by long noncoding RNAs. BiochimBiophysActa.

[8] Li L, Feng T, Lian Y, Zhang G, Garen A, Song X (2009). Role of human noncoding RNAs in the control of tumorigenesis. ProcNatlAcadSci U S A.

[9] Gupta RA, Shah N, Wang KC, Kim J, Horlings HM, Wong DJ, Tsai MC, Hung T, Argani P, Rinn JL, Wang Y, Brzoska P, Kong B, Li R, West RB, van de Vijver MJ, Sukumar S, Chang HY (2010). Long non-coding RNA HOTAIR reprograms chromatin state to promote cancer metastasis. Nature.

[10] Gutschner T, Hämmerle M, Eissmann M, Hsu J, Kim Y, Hung G, Revenko A, Arun G, Stentrup M, Gross M, Zörnig M, MacLeod AR, Spector DL, Diederichs S (2013). The noncoding RNA MALAT1 is a critical regulator of the metastasis phenotype of lung cancer cells. Cancer Res.

[11] Poirier F, Chan CT, Timmons PM, Robertson EJ, Evans MJ, Rigby PW (1991). The murine H19 gene is activated during embryonic stem cell differentiation in vitro and at the time of implantation in the developing embryo. Development.

[12] Tabano S, Colapietro P, Cetin I, Grati FR, Zanutto S, Mandò C, Antonazzo P, Pileri P, Rossella F, Larizza L, Sirchia SM, Miozzo M (2010). Epigenetic modulation of the IGF2/H19 imprinted domain in human embryonic and extra-embryonic compartments and its possible role in fetal growth restriction. Epigenetics.

[13] Berteaux N, Lottin S, Monté D, Pinte S, Quatannens B, Coll J, Hondermarck H, Curgy JJ, Dugimont T, Adriaenssens E (2005). H19 mRNA-like noncoding RNA promotes breast cancer cell proliferation through positive control by E2F1. J Biol Chem.

[14] Berteaux N, Aptel N, Cathala G, Genton C, Coll J, Daccache A, Spruyt N, Hondermarck H, Dugimont T, Curgy JJ, Forné T, Adriaenssens E (2008). A novel H19 antisense RNA overexpressed in breast cancer contributes to paternal IGF2 expression. Mol Cell Biol.

[15] Byun HM, Wong HL, Birnstein EA, Wolff EM, Liang G, Yang AS (2007). Examination of IGF2 and H19 loss of imprinting in bladder cancer. Cancer Res.

[16] Luo M, Li Z, Wang W, Zeng Y, Liu Z, Qiu J (2013). Upregulated H19 contributes to bladder cancer cell proliferation by regulating ID2 expression. FEBS J.

[17] Kim SJ, Park SE, Lee C, Lee SY, Jo JH, Kim JM, Oh YK (2002). Alterations in promoter usage and expression levels of insulin-like growth factor-II and H19 genes in cervical carcinoma exhibiting biallelic expression of IGF-II. BiochimBiophysActa.

[18] Cai X, Cullen BR (2007). The imprinted H19 noncoding RNA is a primary microRNA precursor. RNA.

[19] Gao WL, Liu M, Yang Y, Yang H, Liao Q, Bai Y, Li YX, Li D, Peng C, Wang YL (2012). The imprinted H19 gene regulates human placental trophoblast cell proliferation via encoding miR-675 that targets Nodal Modulator 1 (NOMO1). RNA Biol.

[20] Tsang WP, Ng EK, Ng SS, Jin H, Yu J, Sung JJ, Kwok TT (2010). Oncofetal H19-derived miR-675 regulates tumor suppressor RB in human colorectal cancer. Carcinogenesis.

[21] Kim KI, van de Wiel MA (2008). Effects of dependence in high-dimensional multiple testing problems. BMC Bioinformatics.

[22] Noble WS (2009). How does multiple testing correction work?. Nat Biotechnol.

[23] Ponting CP, Oliver PL, Reik W (2009). Evolution and functions of long noncoding RNAs. Cell.

[24] Wang KC, Chang HY (2011). Molecular mechanisms of long noncoding RNAs. Mol Cell.

[25] Tsang WP, Kwok TT (2007). Riboregulator H19 induction of MDR1-associated drug resistance in human hepatocellular carcinoma cells. Oncogene.

